# Improvement of Liver Fibrosis in Patients with MASLD Undergoing Pioglitazone Treatment: An Update

**DOI:** 10.3390/life15111682

**Published:** 2025-10-29

**Authors:** Cristina Stasi, Andrea Mega

**Affiliations:** 1 Department of Life Science, Health and Health Professions, Link Campus University, 00165 Roma, Italy; 2 Gastroenterology Unit, Bolzano Regional Hospital, 39100 Bolzano, Italy; andrea.mega@sabes.it

**Keywords:** metabolic dysfunction-associated steatotic liver disease, metabolic dysfunction-associated steatohepatitis, fibrosis, cirrhosis, type 2 diabetes, oral antidiabetic agent, pioglitazone, fibrosis improvement

## Abstract

Metabolic dysfunction-associated steatotic liver disease (MASLD) is defined as steatotic liver disease with at least one cardiometabolic risk factor, in the absence of harmful alcohol intake, and includes a spectrum of conditions. These range from isolated liver steatosis to metabolic dysfunction-associated steatohepatitis (MASH), fibrosis, cirrhosis, and MASH-related hepatocellular carcinoma. Patients with MASLD and type 2 diabetes are at increased risk of developing MASH and significant/advanced fibrosis. The severity of fibrosis is a key determinant of long-term prognosis in MASLD. The most recent AASLD and EASL-EASD-EASO Guidelines on the Management of MASLD recommend a step-by-step approach to identify patients at higher risk of fibrotic progression. Recent epidemiological trends highlight the socioeconomic impact of MASLD and MASH, particularly in middle- and low-income countries. Given the high cost of new targeted therapies, implementing effective treatment strategies in low-resource settings is essential in managing MASLD and MASH patients. Pioglitazone is an oral antidiabetic agent of the thiazolidinedione class that targets peroxisome proliferator-activated receptors activated by fatty acids and derivatives or pharmacological agonists and involved in lipid metabolism, cell differentiation, and inflammation. Pioglitazone treatment is a potential cost-effective option, particularly for low-resource settings. This review examines recent epidemiological trends in MASLD and MASH, outlines the mechanisms of action of pioglitazone with an emphasis on its role in improving liver fibrosis, and summarizes clinical studies on fibrosis evaluation during pioglitazone treatment. The literature search focused on English-language studies from the past two years in the PubMed database.

## 1. Introduction

Metabolic dysfunction-associated steatotic liver disease (MASLD), formerly known as Metabolic dysfunction-associated fatty liver disease (MAFLD), is defined by the presence of steatotic liver disease and one or more cardiometabolic risk factors, particularly type 2 diabetes (T2DM) and obesity. MASLD encompasses a spectrum of fatty liver diseases ranging from isolated liver steatosis (metabolic dysfunction-associated steatotic liver, MASL) to metabolic dysfunction-associated steatohepatitis (MASH), as well as fibrosis and cirrhosis and MASH-related hepatocellular carcinoma (HCC) [1].

Subjects with MASLD have a higher risk of developing MASH (31.55%) in the presence of T2DM, developing significant fibrosis (F2–F4) in 35.54% of cases, and advanced fibrosis (F3–F4) in 14.95% [2].

The post-pandemic period has been characterized by important epidemiological changes linked to the direct or indirect impact of COVID-19 [3]. In fact, the COVID-19 pandemic, combined with increased exposure to unhealthy diets and reduced physical activity, led to more people becoming obese [4]. Data from the World Heart Federation [5] highlight that in 2022, 878 million adults worldwide were living with obesity, compared to 194 million living with the disease in 1990.

The severity of fibrosis is an important factor in the long-term prognosis of patients with MASLD, regardless of the underlying cause. Therefore, noninvasive assessment of fibrosis severity is crucial in the management of patients with MASLD. Several non-invasive algorithms based on clinical features and biochemical variables have been tested to identify individuals with MASH and, therefore, at increased risk for adverse clinical outcomes.

Current American Association for the Study of Liver Diseases (AASLD) [6] and European Association for the Study of the Liver, European Association for the Study of Diabetes, European Association for the Study of Obesity (EASL-EASD-EASO) [1] guidelines recommend a step-by-step approach to recognize patients at increased risk of fibrotic progression. The results of a recent study [7] conducted on 7768 adult participants from the National Health and Nutrition Examination Survey 2017–2020 who underwent a valid liver stiffness measurement (LSM) raise concerns about the use of Fibrosis-4 (FIB-4) as an initial test to identify patients at the highest risk of fibrotic progression. In fact, although it showed an excellent specificity (98%), its sensitivity in identifying MASLD or Metabolic and Alcohol-associated liver disease (MetALD) with significant fibrosis (LSM ≥ 8 kPa) was at a low critical level (21%). This poor sensitivity was particularly evident in younger individuals (under 50 years of age) and in those without T2DM 2. Accordingly, Vidal-Trécan et al. [8] enrolled 1572 patients who underwent liver biopsy in the Angiosafe-T2D cohort (NCT02671864; ANR-DGOS 2014), an observational and longitudinal ongoing study. All of the 6 sequential strategies recommended by the EASL-EASD-EASO [1,9], AASLD) [6], American Gastroenterological Association (AGA) [10], American Association of Clinical Endocrinology (AACE) [11], and American Diabetes Association (ADA) [12] foresee the use of FIB-4 as a first-line test, followed by vibration-controlled transient elastography (VCTE). The findings showed that these step-by-step approaches identified 12% of patients who needed hepatology referral. However, considering the 163 patients who underwent liver biopsy, the false negative rates were 15–18% for advanced fibrosis (following the EASL, AGA, and AASLD guidelines). On the contrary, using VCTE as the first step, 19% of patients needed hepatology referrals, with a concomitantly low rate of false negatives (3%) for advanced fibrosis.

The guidelines [1,6,12] include a multidisciplinary approach, encompassing lifestyle modifications, drug therapy, including those for comorbidities such as diabetes, and surgical interventions when appropriate.

Recent epidemiological data highlighted the socioeconomic impact of MASLD and MASH, particularly in middle-and low-income countries, where the burden is expected to increase [13,14]; therefore, in view of more expensive new drugs specifically targeted to these cases, a major effort can begin with implementing therapeutic strategies in low-resource settings to manage such conditions. The EASL-EASD-EASO Guidelines on the Management of MASLD, published in June 2024 [1], consider pioglitazone safe for use in adults with non-cirrhotic MASH. However, due to the lack of robust histological data, it did not yet meet the recommendations as a targeted therapy for MASH. In particular, treatments used for TD2M and obesity, such as pioglitazone and glucagon-like peptide 1 receptor agonists (GLP1RAs), may also have a beneficial effect on the liver. However, pioglitazone, given the insufficient evidence showing fibrosis resolution, cannot be recommended as a targeted therapy for MASH [1]. Furthermore, as strongly recommended by EASL-EASD-EASO [1], GLP1RAs should be used for their respective indications (TD2M and obesity) and are safe in MASH (including compensated cirrhosis), also improving cardiometabolic outcomes. As a strong recommendation by EASL-EASD-EASO [1], resmetirom should be considered for treatment as a MASH-targeted therapy in adults with non-cirrhotic MASH with significant liver fibrosis (stage ≥ 2), as the first FDA-approved MASH treatment for non-cirrhotic patients, because this treatment demonstrated histological efficacy on steatohepatitis and fibrosis with an acceptable safety and tolerability profile. The guidelines obviously note that this recommendation was considered if approved locally, and dependent on the label. The major concern is that not all countries, especially low-income ones, have access to a wide range of therapeutic options for the treatment of MASLD. The AASLD guidelines [6] suggest that pioglitazone improves MASH and can be considered for patients with MASH in the presence of T2DM.

According to these premises, this review provides an overview of the epidemiological changes in MASLD and consequently MASH; the mechanisms of action of pioglitazone, particularly its effects on inflammation and liver fibrosis; and finally, clinical studies evaluating the improvement in liver fibrosis during pioglitazone treatment. This search was conducted using the PubMed database for relevant studies published in English from 1 May 2024 (see Section 4).

In accordance with the latest guidelines [1], this review will utilize the most recent nomenclature of MASLD and MASH.

## 2. The Burden of MASLD and MASH

Epidemiological data showed a progressive increase in MASLD from 25.3% in 1990–2006 to 38.0% in 2016–2019, with a higher prevalence in males compared to females [14]. Recently, Guo et al. [13], based on Global Burden of Disease (GBD) 2021 data, reported 1,267,867,997 global cases of MASLD, with a significantly increasing trend for the age-standardized prevalence rate of MASLD, MASLD-related cirrhosis, and MASH-related liver cancer. In parallel, the incidence showed a significant increase in these conditions. In this context, in 2021, South Asia exhibited the highest number of incidences of MASLD and MASLD-related cirrhosis, and East Asia that of MASH-related liver cancer. A significant increasing trend was also found in global deaths in 2021 vs. 1991 for MASLD (138,328 vs. 59,536), MASLD-related cirrhosis (97,403 vs. 44,861), MASH-related Liver Cancer (40,925 vs. 14,675), with the highest mortality rate in people aged over 95 years, followed by those between 90 and 94 years and then 85–89 years, without significant gender differences across age classes.

Danpanichkul et al. [15] found that MASH-related primary liver cancer in young adults has increased significantly. Indeed, analyzing cases between 2000 and 2021, MASH-related primary liver cancer in young adults had an incidence of 6%, resulting in a 1% increase in incident cases (since 2000), as well as a 6% increase in deaths (+2% since 2000), a 6% increase in disability-adjusted life years (+2% since 2000) for all primary liver cancers in young adults.

Younossi et al. [16] conducted a meta-analysis of 123 studies (2,241,753 patients with T2D), finding a global MASLD prevalence among T2D patients of 65.33% (436 million people). The prevalence increased from 55.86% (1990–2004) to 68.81% (2016–2021), with Eastern Europe showing the highest prevalence. Among 1002 liver biopsies in MASH patients, 66.44% had MASH, 40.78% significant fibrosis, and 15.49% advanced fibrosis. All-cause mortality was 16.79 per 1000 person-years, with liver-specific mortality at 2.15 per 1000 person-years. Another meta-analysis by Wongtrakul et al. [17] showed an association between MASLD and increased all-cause mortality in T2DM patients, supporting MASLD screening.

A recent study by Younossi et al. [18] evaluated the impact of MASH on clinical, economic, and quality of life outcomes. Findings indicated significant clinical burden, increased healthcare costs, and notable reductions in patient quality of life as the disease progressed from early to advanced stages. Using the Markov model, this impact was evaluated by simulating disease progression annually from 2021 to 2040 across nine countries. The baseline cohort was stratified into fibrosis stages (F0–F3), compensated cirrhosis, decompensated cirrhosis, hepatocellular carcinoma, liver transplant, and post-liver transplant. The overall prevalence of MASH in 2020 ranged from 0.23% in Japan to 0.82% in the US; in Italy, it was 0.41%. In the US, the distribution of liver fibrosis stages based on data from Estes et al. [19] and Alswat et al. [20] showed 18.36% in F0, 36.30% in F1, 21.49% in F2, 13.88% in F3, and 9.96% in F4. By 2040, MASH prevalence is expected to increase from 6.71% to 7.41% in the U.S, 4.58% to 5.37% in Italy, and 7.19% to 7.52% in Brazil. The prevalence rate of advanced MASH (including F3, cirrhosis, HCC, and Liver transplants) is expected to rise by ≥20% in all countries. Direct annual medical costs are projected to more than double ($34.97 to $78.59 billion in the U.S., $1.34 to $3.00 billion in Italy, $3.41 to $9.81 billion in Brazil). At the same time, losses in work productivity are expected to more than double in most countries, and health-related quality of life is projected to decline as the burden of advanced diseases increases [18].

## 3. Mechanism of Action of Pioglitazone and Its Anti-Fibrotic Effects

Patients with MASLD can be classified into three phenotypes: those with a hepatic genetic component, who have a moderate to high risk of MASH and fibrosis, those with a metabolic component related to hepatic de novo lipogenesis, and those with a metabolic component related to adipose tissue dysfunction, all of whom have a moderately increased risk of MASH and fibrosis [21]. The pathogenesis of MASLD has been extensively documented [21,22]. One of the “multiple hits” responsible for the transition to MASH is lipotoxicity, resulting in selective activation of cell death pathways, mitochondrial dysfunction and oxidative stress, and activation of inflammatory signaling pathways, with subsequent hepatocellular injury and activation of hepatic stellate cells (HSCs) and extracellular matrix deposition, leading to fibrosis [23].

Pioglitazone, an oral antidiabetic agent in the thiazolidinedione class, targets insulin resistance by activating peroxisome proliferator-activated receptors (PPARs). The PPAR subfamily consists of three PPAR isotypes, encoded by distinct genes: PPARα (NR1C1), PPARß/δ (also called NR1C2), and PPARγ (NR1C3) [24]. These receptors exhibit different expression patterns across organs and contribute in complementary ways to the pathogenesis of MASH [25]. The PPARγ targets insulin resistance by promoting peripheral insulin sensitivity, enhancing glucose uptake by skeletal muscle, suppressing liver release of glucose into the bloodstream, and improving the secretory response of insulin by pancreatic β-cells [26].

PPARγ inhibits liver inflammation in hepatocytes and expression of pro-inflammatory mediators such as cyclooxygenase-2, interleukin (IL)-6, tumor necrosis factor-α (TNF-α), nitric oxide, and reactive oxygen species (ROS) [27]. This suppression reduces transcription of pyroptosis pathways, including inflammasome 3 (NLRP3) [28]. Pyroptosis is characterized by the formation of gasdermin D pores in cell membranes following inflammatory cell death with subsequent release of pro-inflammatory cytokines [23].

The crucial aspect of chronic liver diseases is their potential for fibrosis progression, which occurs in a dynamic context where the accumulation of fibrillar extracellular matrix (ECM) is linked to an imbalance between degradation and remodeling of the matrix, mediated by the matrix metalloproteinases (MMPs) and their inhibitors (TIMPs) [29]. In vitro, pioglitazone prevented the activation of HSC by reducing the expression of type I procollagen, MMP-2, TIMP-1, and TIMP-2 mRNA with increased MMP-13 mRNA expression [30]. Deng et al. [31] evaluated the effects of pioglitazone in MASLD and investigated the underlying mechanism by testing platelet-derived growth factor (PDGF) and tissue inhibitor of metalloproteinase-2 in 180 C57BL/6 wild-type mice equally distributed in 3 groups: control group, high-fat control group, and pioglitazone treatment group. Findings highlighted that expressions of PDGF and tissue inhibitor of metalloproteinase-2 proteins in serum were reduced in MASLD mice in response to pioglitazone treatment. In addition, liver histology demonstrated an inhibitory effect of pioglitazone treatment on steatosis and inflammation, showing a small number of bullous fatty degeneration and absence of hepatocyte necrosis and inflammatory cell infiltration, which in turn suppresses the subsequent hepatic fibrosis.

Apart from pioglitazone’s regulatory properties on metalloproteases, its activity on cytokine suppression can inhibit TGF-β signaling [28]. The activation of HSCs toward a pro-fibrogenic myofibroblast type is primarily mediated by TGF-β, and activation of PPARγ upregulates hepatocyte growth factor (HGF) transcription, promoting hepatocyte proliferation. In fact, PPRγ expression decreases with conversion to myofibroblasts, while PPAR γ agonism reduces HSC activity [25]. Miyahara et al. [32] evaluated the ability of PPAR to activate HSC of rats from cholestatic liver fibrosis induced by bile duct ligation. In culture-activated HSC, the ligands for PPARγ at the concentrations that activate PPR-γ consistently reversed biochemical changes that were characteristic of HSC activation. This study demonstrated that PPARγ ligands inhibited collagen gene expression, in part, by suppressing collagen gene promoter activity. Oxidized low-density lipoprotein is considered to contain PPARγ ligands, and once internalized, it inhibits the HSC-activated phenotype. On the other hand, this inhibition may also be opposed by the effects of lipid peroxides and aldehydes liberated from oxidized low-density lipoprotein, which induce oxidative stress and signal for activation of HSC. Moreover, PPARγ ligand-induced up-regulation of a fatty acid transporter CD36, may enhance the uptake of long-chain fatty acids, potentially promoting lipid storage. These findings demonstrate that HSC activation is associated with the reduction in PPR-γ expression and binding in vivo and is reversed by the treatment with PPR-γ ligands in vitro.

Based on the effects of pioglitazone, the next section moves from the bench to the patient’s bedside.

## 4. Clinical Evaluation of Liver Fibrosis Improvement During Pioglitazone Treatment

### 4.1. Methodology of Literature Review

A structured literature search of clinical studies using the PubMed database was conducted from 1 May 2025, to the present (15 October 2025). The original research studies involving human subjects were eligible for inclusion if they evaluated liver fibrosis through direct or indirect serum markers or liver biopsy during pioglitazone treatment, and if they were published within two months before the date of publication of the EASL-EASD-EASO Clinical Practice Guidelines on the management of metabolic dysfunction-associated steatotic liver disease [1]. These guidelines were considered the gold reference standard for the strong evaluation of previously published studies.

In addition, studies were eligible if they were peer-reviewed, published in English, and met the inclusion criteria.

The search strategy in the index period includes the following items: “pioglitazone and liver fibrosis” (first item), “pioglitazone and MASH” (second item), “pioglitazone and MASLD” (third item), “pioglitazone and NAFLD” (fourth item), “pioglitazone and NASH” (fifth item).

Exclusion study designs included reviews, systematic reviews, meta-analyses, clinical guidelines, and expert consensus statements.

When we searched Clinical Trials.gov for the same items and study completion period, we found no trials.

**Figure 1:**
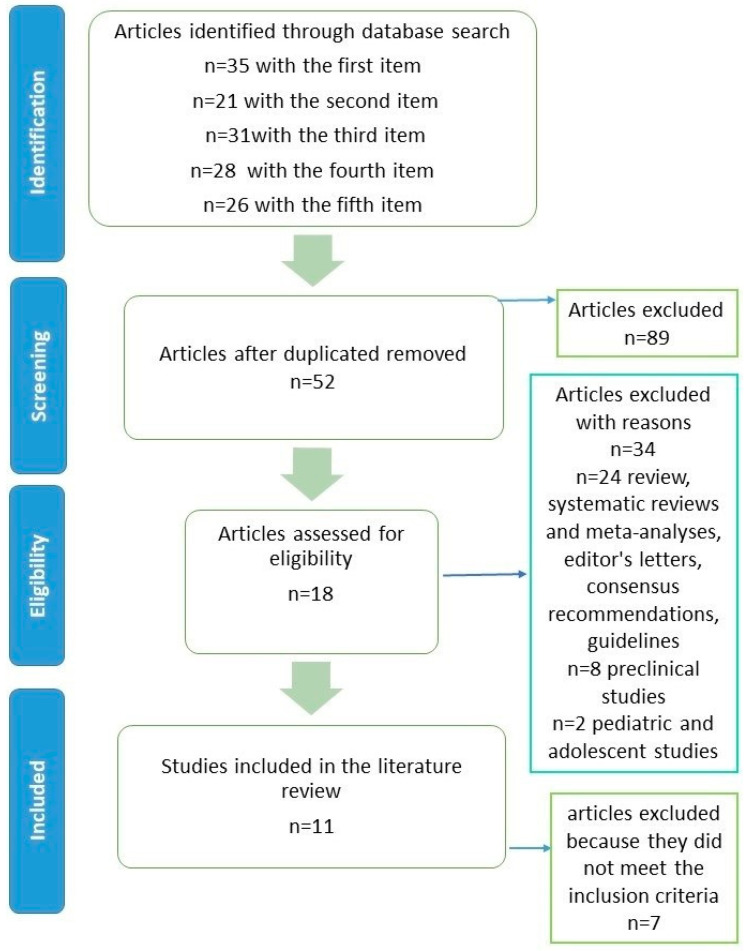
Flow diagram for the literature review.

Duplicates were removed (*n* = 89). 52 unique articles remained. Of these, 11 met the inclusion criteria (Figure 1).

### 4.2. Literature Review

This section reviews recent studies in adults using pioglitazone, either alone or in combination, and assesses liver fibrosis using particularly non-invasive methods, given the scarcity of recent biopsy data. Among previous studies, Gawrieh et al. [33] assessed the Enhanced Liver Fibrosis Score (ELF) and amino-terminal propeptide of type III procollagen (PIIINP) in relation to fibrosis stages at baseline and after treatment with vitamin E or pioglitazone in the PIVENS study. Over 96 weeks, the ELF decreased significantly with vitamin E but not with pioglitazone. Changes in ELF did not correlate with improvement in fibrosis or MASH resolution. However, PIIINP levels correlated significantly with MASH resolution and improved MASLD activity scores, decreasing in both the vitamin E and pioglitazone groups compared to the placebo group.

Table 1 summarizes the fatty liver and liver fibrosis outcomes after monotherapy or combination treatment with pioglitazone during a follow-up time < 1 year conducted in the index period and fails the items for search. To use the new terminology, the MASLD fibrosis score has also been replaced with the term MASLD fibrosis score.

The study by Abdel Monem et al. [34] evaluated the efficacy and safety of dapagliflozin in 50 diabetic and 50 non-diabetic biopsy-proven MASH patients compared to pioglitazone. After 24 weeks, MASLD activity score and liver steatosis assessed by biopsy significantly improved in non-diabetic patients treated with dapagliflizin, in diabetic patients treated with dapagliflozin, and in diabetic patients treated with pioglitazone. The MASLD fibrosis score significantly improved among non-diabetic patients treated with dapagliflozin, and FIB-4 improved in both non-diabetic and diabetic patients treated with dapagliflozin. Both MASLD fibrosis score (−1.04 ± 1.28 vs. −0.56 ± 1.14, *p* = 0.016) and FIB-4 (1.08 ± 0.48 vs. 1.40 ± 0.72, *p* = 0.015) scores showed significant negative changes in diabetic patients treated with pioglitazone and non-significant changes in non-diabetic patients treated with pioglitazone. Significant differences in ΔMASLD fibrosis score and ΔFIB-4 were found in diabetic patients treated with dapagliflozin. FibroScan results showed greater improvement in both fibrosis and steatosis in the dapagliflozin group compared to the pioglitazone group. Only in the group of non-diabetic patients treated with dapagliflozin was there a significant within-group improvement in the grades of fibrosis and steatosis.

In 2020–2021, Sayadishahraki et al. [35] conducted a randomized clinical trial on 44 patients with grade 3 MASLD (ultrasound examinations), and candidates for bariatric surgery. Of these, 22 patients were assigned to treatment with pioglitazone 15 mg tablets twice daily for 4 months, and 22 in the control group were given a special diet. After four months of treatment with pioglitazone, 50% of patients showed grade 1 MASLD, while the other 50% patients showed grade 2 MASLD, with significant improvements in liver size, left liver lobe size, fasting blood glucose, alanine aminotransferase, and BMI. No data on liver fibrosis were reported. The control group showed no significant improvements.

Lee et al. [36] evaluated the effects of combination therapy with pioglitazone (15 mg/d) and empagliflozin (10 mg/d) in MASLD patients with T2DM compared with pioglitazone 15 mg alone, or empagliflozin 10 mg alone. Patients with a ≥30% relative reduction or ≥5% absolute reduction in liver fat were most common in the combination group (100.0% vs. 57.1% for pioglitazone and 87.5% for empagliflozin). Combination therapy also induced the highest percentage of individuals with a ≥30% relative reduction in liver fat and ≥20% relative reduction in liver stiffness compared to the monotherapy groups.

Khaliq et al. [37] evaluated the effects of ertugliflozin 15 mg oral tablet versus pioglitazone 30 mg in 180 patients with MASLD and T2DM randomized into three groups (ertugliflozin, pioglitazone, placebo). The finding demonstrated a marked decrease in body weight in the ertugliflozin and pioglitazone group. AST, ALT, and ALP, TG, LDL, and Cholesterol levels decreased in the drug-treated groups, but ALT and AST levels decreased more in the ertugliflozin group, while the ALP level decreased significantly in the pioglitazone group. The BMI decreased in the drug-treated groups, but the ertugliflozin group reached a significantly lower BMI compared to the pioglitazone group. The FIB-4 index ratio (1.82 ± 0.46 at baseline vs. 1.04 ± 0.43 after 24 weeks of ertugliflozin treatment) and insulin resistance HOMA-IR levels (3.99 ± 0.53 vs. 3.10 ± 0.45) significantly decreased.

**Table 1:** Some liver features after monotherapy or combination treatment with pioglitazone during a follow-up time < 1 year.

Authors	Study Design	Country	Number of Patients	Study Population/Treatment	Follow-Up Time <1 Year	Liver Steatosis/ Methods	Liver Fibrosis/ Non-Invasive Methods	Biopsy
Abdel Monem et al. [34]	Prospective, randomized, parallel, open-label study	Egypt	100	100 patients with MASH biopsy-confirmed, diabetic and non-diabetic, randomized to pioglitazone 30 mg/day vs. dapagliflozin 10 mg/day	24 weeks	Dapagliflozin showed a comparable effect on improvement in liver steatosis grade assessed by transient elastography from baseline in diabetics versus a significant superiority in non-diabetics	Dapagliflozin showed a comparable effect on improvement in liver fibrosis stage assessed by transient elastography from baseline in diabetics versus a significant superiority in non-diabetics	Dapagliflozin and pioglitazone had a comparable effect on changes in the MASLD activity score in both the non-diabetic and diabetic groups (*p* > 0.05), with the exception of Δ hepatocellular ballooning in favor of dapagliflozin in the diabetic group (*p* = 0.048). The improvement from baseline for non-diabetic patients was not significantly higher in the dapagliflozin group compared to the pioglitazone group.
Lee et al. [36]	Randomized, open-label trial	Korea	50	50 patients with TD2M and MASLD randomized to pioglitazone 15 mg/die vs. empaglifozin 10 mg/die vs. pioglitazone plus empaglifozine	24 weeks	Combination therapy resulted in the largest reduction in liver fat assessed by magnetic resonance imaging-proton density fat fraction	Combination therapy resulted in the largest reduction in liver stiffness assessed by magnetic resonance elastography	
Khaliq et al. [37]	prospective, randomized, double-blind, placebo-controlled, interventional study	Pakistan Malaysia	180 completed the trial	180 MASLD and TD2M patients with fatty liver from grade 1 to grade 3, (ertugliflozin, *n* = 60; pioglitazone, *n* = 60; placebo, *n* = 60)	Final FU at 24 weeks	In the ertugliflozin and pioglitazone groups, 45% to 23.4% and 41.7% to 26.6%, respectively, decreased in the grade 2 group assessed by ultrasonography	FIB-4 index decreased markedly after ertugliflozin treatment.	-
Khaliq et al. [38]	double-blind, randomized, controlled clinical trial	Pakistan	173 patients	173 patients with MASLD and T2DM randomized to receive vitamin E 800 IU daily (*n* = 42), pioglitazone 30 mg daily (*n* = 43), ertugliflozin 15 mg daily (*n* = 44), and vitamin E 800 IU daily + ertugliflozin 15 mg daily (*n* = 44) combination therapy	24 weeks	Fatty liver grades markedly decline in the group that received vitamin E combined with ertugliflozin	FIB-4 reduction during treatment with ertugliflozin + vitamin E. Pioglitazone and vitamin E showed no effect on the FIB-4.	

Abbreviations: T2DM: type 2 diabetes; HbA1c: Hemoglobin A1c; MASLD: Metabolic dysfunction-associated steatotic liver disease; FIB-4: Fibrosis-4; FAST™: FibroScan-AST; MASH: metabolic dysfunction-associated steatohepatitis; BW: body weight.

Kaliq et al. [38] evaluated 173 patients with MASLD and T2DM, assigned into four groups: vitamin E (*n* = 42), pioglitazone (*n* = 43), ertugliflozin (*n* = 44), and vitamin E+ertugliflozin (*n* = 44) to evaluate insulin resistance, oxidative stress, and inflammation. After 24 weeks of treatment, the study showed a significant amelioration of metabolic parameters (blood sugar levels, HbA1C, HOMA-IR, BMI) in the ertugliflozin, pioglitazone, and vitamin E+ertugliflozin groups (*p* < 0.001), where vitamin E did not reach them. Transaminase levels significantly improved in all groups. Ertugliflozin markedly reduced FIB-4, especially when combined with vitamin E, while pioglitazone and vitamin E showed no effect on FIB-4. Although all treatments were effective in reducing fatty liver severity, the combination therapy of vitamin E and ertugliflozin had the most pronounced efficacy, followed by ertugliflozin monotherapy.

Table 2 summarizes the fatty liver and liver fibrosis outcomes after monotherapy or combination treatment with pioglitazone during a follow-up time > 1 year conducted in the index period and fails the items for search. Abdul-Ghani et al. [39] evaluated the effects of two therapeutic approaches: sequential therapy, initiating with metformin followed by the addition of sulfonylurea and insulin, and initial therapy with metformin plus pioglitazone plus exenatide. The study endpoints included changes in liver fat content, liver fibrosis, and carotid intima–media thickness, measured over six years in the Efficacy and Durability of Initial Combination Therapy for Type 2 Diabetes (EDICT) study, which included 55 TD2M patients. The results showed excellent glycemic control (HbA1c < 6.5%) in all participants during follow-up, along with improvements in blood pressure and LDL cholesterol. However, patients on standard therapy exhibited continued progression in the primary endpoints—carotid intima–media thickness, steatosis, and liver fibrosis—compared to those receiving initial triple therapy (metformin plus pioglitazone plus GLP-1 RA) (Table 2).

Albert et al. [40] evaluated 220 adults with MASLD who did not have diabetes mellitus to evaluate whether FIB-4 can effectively screen and monitor changes. Participants were randomized to receive pioglitazone, vitamin E, or a placebo. All underwent liver biopsy, and the MASLD activity score and FIB-4 were calculated. These examinations were repeated at 96 weeks in 220 patients. In the pioglitazone group, the NAS score decreased by 39% (4.78 ± 1.36 vs. 2.93 ± 1.55). In the vitamin E group, NAS decreased by 36% (5.05 ± 1.36 vs. 3.22 ± 1.42). These changes were correlated with changes in FIB-4 scores. Steatosis decreased by 43% and 38%, and inflammation by 37% and 35% in the pioglitazone and vitamin E groups, respectively. As demonstrated in other studies [41], antidiabetic drugs are also effective in MASLD patients without TD2M and probably in a pre-diabetic state.

In an open-label study, Ito et al. [42] evaluated 66 patients with MASLD with TD2M, randomly assigned to receive 50 mg of ipragliflozin once daily (*n* = 32) or 15–30 mg of pioglitazone once daily (*n* = 34) for 24 weeks. Of these patients, 61 were followed for 5 years glycemic and metabolic parameters, as well as MASLD fibrosis, FIB-4, and type IV collagen 7S values (NAFIC) scores. Patients were defined as having suspected progression to MASH if they had a MASLD fibrosis score ≥0.675, FIB-4 index ≥2.67, or NAFIC score ≥2 points. The results demonstrated that ipragliflozin and pioglitazone significantly improved hepatic fat accumulation, serum transaminase levels, and glycemic parameters during 5 years. Furthermore, in the ipragliflozin group, significant reductions in body weight and visceral fat mass persisted for 5 years. In patients with suspected progression to MASH, both ipragliflozin and pioglitazone maintained improvements in MASLD/MASH parameters for 5 years. However, a significant reduction in serum ferritin levels and FIB-4 index was observed only in the ipragliflozin group.

Out of our search results, because published in 2023, Hooshmand Gharabagh et al. [43] compare the efficacy of combination therapy of empagliflozin (10 mg/day) or pioglitazone (30 mg/day) with metformin in 60 patients with T2DM and MASLD, evaluating MASLD grade and liver steatosis and fibrosis, anthropometric characteristics, lipid profile, plasma glucose, and liver enzymes. Findings demonstrated significant regression of liver fibrosis, lipid profiles, and liver enzymes with both combination therapies. However, the greatest reduction in waist circumference was observed in the empagliflozin-treated groups compared to those treated with pioglitazone. Weight and BMI decreased significantly only in patients receiving empagliflozin.

Pereira et al. [44] retrospectively evaluated 65 patients with MASLD undergoing pioglitazone therapy at three public hospitals in the southwestern region of Brazil, investigating the long-term effects of therapy on liver stiffness, steatosis, and non-invasive biomarkers. Patients received treatment with Pioglitazone (30–45 mg) when MASLD was associated with T2DM or in cases of fibrosis stage ≥F2 assessed by liver biopsy and they were longitudinally assessed from baseline to the end of follow-up with the FAST™ score, combining FibroScan^®^ parameters such as LSM and Controlled Attenuation Parameter (CAP) with Aspartate Aminotransferase (AST). This score was validated by Newsome et al. [45]. When patients were stratified into two groups based on treatment duration (1–3 and 4–10 years), gamma glutamyl transpeptidase were significantly decreased only in the first group (1–3 years); alanine aminonotransferase and FAST score in both groups; HbA1c (%), Serum Glucose, CAP only in the second group (4–10 years). The improvement in liver enzymes, non-invasive liver fibrosis scores, liver steatosis, and glycemic control over time supports the use of this treatment for MASLD. They found a significant reduction in FAST™ score between baseline and the 1–3 years follow-up period (*p* = 0.042), which markedly improved in 4–10 years period (*p* = 0.012) with a concomitant CAP reduction in the same follow-up period, suggesting that the steatosis and liver fibrosis occur in long term treatment period, compared to the reversal of necroinflammation which occurs in a shorter period. The authors [44] also emphasized that, although the improvement may be modest compared to newer agents, the cost-effectiveness and metabolic and liver benefits make pioglitazone a viable option for a broader population.

**Table 2:** Some liver features after monotherapy or combination treatment with pioglitazone during a follow-up time > 1 year.

Authors	Study Design	Country	Number of Patients	Study Population/ Treatment	Follow-Up Time >1 Year	Liver Steatosis/ Methods	Liver Fibrosis/ Methods	Biopsy
Abdul-Ghani et al. [39]	Clinical Trial (Trial registration number NCT01107717)	USA	55	T2DM patients received initial triple therapy with metformin/pioglitazone/exenatide (*n* = 29) vs. metformin, followed by stepwise addition of glipizide and then insulin glargine (*n* = 26), achieving glycemic control (HbA1c < 6.5%)	6 years	Attenuation of progression of liver steatosis assessed by controlled attenuation parameter in patients receiving initial triple therapy	Attenuation of the progression of liver fibrosis assessed by transient elastography in patients receiving initial triple therapy	
Albert et al. [40]	Prospective study	USA	220	220 patients with MASLD without diabetes mellitus (pioglitazone vs. vitamin e vs. placebo).	96 weeks	Pioglitazone and vitamin E both improved histological steatosis	MASLD activity scores improved with pioglitazone by 39%, and with vitamin E by 36%, correlating with changes in FIB-4	Improvement of steatosis, and inflammation, no change in fibrosis stage
Ito et al. [42]	Randomized active-controlled trial	Japan	61	MASLD and T2DM patients were randomly assigned to receive either ipragliflozin (*n* = 31) or pioglitazone (*n* = 30)	24 weeks 5 years	Improvement of liver steatosis, as evaluated using the liver-to-spleen attenuation ratio (L/S ratio) on computed tomography for both ipragliflozin and pioglitazone	FIB-4 index significantly improved only in the ipragliflozin group	
Pereira et al. [44]	Multi-center retrospective study	Brazil	65	MASLD patients treated with pioglitazone (30–45 mg) when MASLD was associated with T2DM or fibrosis stage ≥F2 assessed by liver biopsy	1–3 years 4–10 years	Significant reduction in CAP levels only in the 4–10 follow-up period, suggesting a reduction in liver steatosis	Significant reduction in FAST™ score between baseline and two follow-up periods	
Martínez-Sanchez et al. [46]	Multicentric retrospective observational study	Mexico	2000	2000 patients with T2DM stratified for FIB-4 values (*n* = 935 in F0, 658 in F1–F2, 407 in F3–F4)	Median duration of T2D = 7 years		Statins and pioglitazone may protect against liver fibrosis	
Shi et al. [47]	Multicenter retrospective cohort study	China Korea United Kingdom Italy Japan Sweden Malaysia France Singapore Virginia Spain Massachusetts	7867	7867 patients with T2D and MASLD Treated with pioglitazone, GLP-1RAs, and SGLT-2 inhibitors	5.1 years		SGLT-2 inhibitor had a significantly lower risk of liver stiffness progression	At baseline, 1599 patients underwent liver biopsy, of whom 42.4% had advanced fibrosis and 71.3% had MASH
Lee et al. [48]	Real-world prospective cohort study	China	888	888 patients with T2D and MASLD Treated with pioglitazone, GLP-1RAs, and SGLT-2 inhibitors	3.9 years	*	Participants had received a median of three reassessment VCTEs and their FAST * scores overall improved significantly from baseline. Dual or triple combination treatment was significantly associated with a greater annual reduction in FAST scores from baseline	

Abbreviations: T2DM: type 2 diabetes; HbA1c: Hemoglobin A1c; MASLD: Metabolic dysfunction-associated steatotic liver disease; FIB-4: Fibrosis-4; FAST™: FibroScan-AST; MASH: metabolic dysfunction-associated steatohepatitis; VCTE: vibration-controlled transient elastography. * FAST scores were calculated using an equation that consisted of AST, CAP and LS values.

Martínez-Sanchez et al. [46], using medical records from four centers in Mexico City from 2018 to 2023, studied factors associated with advanced liver fibrosis, assessed by FIB-4, in 2000 patients with T2DM. The study also evaluated clinical characteristics and biochemical variables among different liver fibrosis subgroups. Clinical nad biochemical data of patients were stratified for FIB-4 values (*n* = 935 in F0, 658 in F1–F2, 407 in F3–F4). The median duration of T2D was 7 years, and the mean HbA1c was 7.63%. The patients group with F < 2 were younger, women, had lower waist circumference, shorter duration of TD2M, higher use of metformin, pioglitazone, any fibrates and statins, lower use of subcutaneous insulin, lower fasting plasma glucose, serum creatinine, microalbuminuria and higher serum sodium levels, INR, total cholesterol, LDL-C, triglycerides, GGT, and serum albumin. The findings of this study demonstrated that longer duration of diabetes, hypolipidemia, insulin resistance, and microalbuminuria were associated with advanced liver fibrosis. On the contrary, pioglitazone (OR = 0.111 [95% CI 0.073–0.168]) and statins (OR = 0.082 [95% CI 0.010–0.672]) showed lower odds of advanced liver fibrosis, suggesting a protective effect of these drugs. This suggests that pioglitazone is likely to demonstrate long-term beneficial effects in real practice with personalized therapy.

Shi et al. [47] evaluated the long-term effects of pioglitazone, GLP-1RA, and SGLT-2 inhibitors on the risk of developing hepatic events and progression of liver stiffness in 7867 patients with TD2M and MASLD. The incidence rate of hepatic events was significantly lower in the group treated with SGLT-2 inhibitors compared to the group not treated with this treatment. However, no significant associations were observed between pioglitazone, GLP-1RA, and SGLT-2 inhibitors and the risk of all-cause mortality. In addition, the rate of liver stiffness progression was significantly lower in the SGLT2 inhibitor group than in those without this treatment.

Lee et al. [48] studied longitudinal changes in FAST scores in 888 patients with TD2M and MASLD. During a median follow-up of 3.9 years, FAST scores improved overall from baseline (*p* < 0.001), in parallel with a reduction in body weight and HbA1c. The combination of these antidiabetic agents (double or triple combination) was independently associated with a higher likelihood of a FAST score ≤ 0.35 (OR 2.40, 95% CI 1.11–5.21, *p* = 0.026).

Although some of these studies showed no resolution of fibrosis or steatohepatitis from baseline to follow-up, they demonstrated improvement in fibrosis and clinical parameters, suggesting a clinical benefit in initiating treatment in the early stages of steatohepatitis, before the onset of advanced fibrosis.

Currently, a recruiting single-center phase 2A, randomized, double-blind, placebo-controlled study is evaluating the safety and efficacy of low-dose pioglitazone (15 mg per day) on liver histology in patients aged 21 to 75 years with T2DM biopsy-proven MASH [49].

Markowska et al. [50], among the botanical ingredients belonging to the flavonoid group, summarized antioxidant, anti-inflammatory, and lipid-regulating properties of quercetin, suggesting it as a complementary treatment in MASLD patients, due to its beneficial effects. In this regard, the study by Kunasegaran et al. [51] demonstrated that a combination of quercetin and pioglitazone prevents T2DM and reduces oxidative stress.

Regarding the safety profile of pioglitazone, a recent meta-analysis [52] evaluated the adverse effects of pioglitazone and sodium–glucose transporter-2 (SGLT2) inhibitors in Asian patients with MASLD and T2DM as a secondary outcome. The adverse event analysis was conducted on eight RCTs involving 637 patients, with low heterogeneity observed. The results showed that there were no significant differences in the likelihood of adverse events between the pioglitazone and SGLT2 inhibitor groups (RR 1.28, 95% CI 0.83 to 1.96, *p* = 0.26) [52]. However, a meta-analysis [53] including nine trials with 12,026 participants demonstrated that pioglitazone was associated with several significant adverse effects, including heart failure (RR 1.32; CI 1.14 to 1.54), bone fracture (RR 1.52, 95% CI 1.17 to 1.99), edema (RR, 1.63; CI 1.52 to 1.75) and weight gain (RR 1.60; CI 1.50 to 1.72). In addition, a systematic review and meta-analysis by Tanq et al. [54] reported that in observational studies, the slightly increased risk of bladder cancer among ever-users of pioglitazone versus never-users (OR, 1.13; 95%CI, 1.03 to 1.25), which was time- and dose-dependent.

A Brazilian study [55] using Bayesian networks evaluated the efficacy and cost-effectiveness of antihyperglycemic therapies in patients with T2DM. In 2023, the Brazilian Unified Health System did not include pioglitazone, SGLT2 inhibitors, or GLP-1A in its portfolio of antihyperglycemic drugs; the authors calculated the combined cost by considering Brazilian Unified Health System expenditures together with out-of-pocket expenditures. The study found an association between pioglitazone, SGLT2 inhibitors, and GLP-1A and reduced risk of major cardiovascular events. In addition, the results showed that SGLT2 inhibitors and GLP-1As reduced cardiovascular mortality, and pioglitazone increased the incidence of hospitalization for heart failure. However, the results demonstrated a greater likelihood of greater cost-effectiveness with pioglitazone, followed by SGLT2 and GLP-1A inhibitors. Specifically, SGLT2 inhibitors and pioglitazone were highly likely to be cost-effective in both primary prevention (asymptomatic patients, starting follow-up at age 47 years) and secondary prevention scenarios (follow-up at age 60 years, corresponding to the mean age of first cardiovascular or cerebrovascular event or first hospital admission) using estimated treatment costs in Brazil.

Based on the results of these recent studies, it is evident that pioglitazone can slow down the evolution of steatosis and fibrosis in monotherapy, with a potentiated effect on the improvement in fibrosis in combination therapies. This drug could probably be used in specific categories of patients and contexts, taking precautions based on the safety profile, with the necessary monitoring procedures.

## 5. Conclusions

Studies vary widely regarding pioglitazone dosage and combination therapy. Most assess fibrosis using indirect serum markers, while some rely on liver biopsy. The length of follow-up also varies, but longer studies consistently report improvement in liver fibrosis. This likely reflects the multifactorial nature of MASLD, in which all contributing factors must be addressed before fibrosis improvement.

To accurately assess the efficacy of long-term treatment, short-term risk factors for necroinflammation must be assessed. Although further research is needed for patients with MASH and prediabetes, recent evidence supports the efficacy of pioglitazone monotherapy and its benefit in improving liver fibrosis in combination therapy for MASH.

The pioglitazone treatment shows the ability to treat steatohepatitis and improve insulin sensitivity while acknowledging its potential use as a secondary treatment for diabetic MASH patients, but also its inability to resolve fibrosis. Resmetirom and GLP-1/GIP receptor agonists are changing treatment options for MASLD by showing better performance than pioglitazone in both safety and effectiveness. Future studies should evaluate the cost-effectiveness of treating patients with oral antidiabetics, specifically those with MASLD and T2DM, keeping in mind that cost-effectiveness models vary greatly depending on the national context. The cost of new drugs offers clinical benefits and fewer adverse effects, but in some cases, they are “out of range” for low-income countries. As rates of MASLD continue to increase globally, particularly in low- and middle-income countries due to rising obesity and T2DM, pioglitazone may represent a viable option where access to newer, more expensive medications is limited.

## Data Availability

Data supporting reported results can be found in published articles available in PubMed, specifically at references [34,36,37,38,39,40,42,44,46,47,48].

## Abbreviations

The following abbreviations are used in this manuscript:

MASLD	Metabolically-dysfunction-associated steatotic liver disease
MAFLD	Metabolically-dysfunctional-associated fatty liver disease
T2DM	type 2 diabetes
MASL	Metabolically-dysfunction-associated steatotic liver
MASH	Metabolically-dysfunction-associated steatohepatitis
HCC	Hepatocellular carcinoma
AASLD	American Association for the Study of Liver Diseases
EASL-EASD-EASO	European Association for the Study of the Liver, European Association for the Study of Diabetes, European Association for the Study of Obesity
LSM	Liver stiffness measurement
FIB-4	Fibrosis-4
MetALD	Metabolic and alcohol-associated liver disease
AGA	American Gastroenterological Association
AACE	American Association of Clinical Endocrinology
ADA	American Diabetes Association
VCTE	Vibration-controlled transient elastography
HCSs	Hepatic stellate cells
PPARs	Peroxisome proliferator-activated receptors
TNF-α	Tumor necrosis factor-α
ROS	Reactive oxygen species
NLRP3	Inflammasome 3
ASC	Apoptosis-associated speck-like protein containing a caspase recruitment domain
Nrf2	Nuclear factor erythroid 2-related factor 2
HO-1	Heme oxygenase-1
HGF	Hepatocyte growth factor
PDGF	Platelet-derived growth factor
HbA1c	Hemoglobin A1c
FAST™	FibroScan-AST
NAFIC	Type IV collagen 7S values
CAP	Controlled Attenuation Parameter
AST	Aspartate Aminotransferase
